# Harnessing biodiversity: the Malagasy Institute of Applied Research (IMRA)

**DOI:** 10.1186/1472-698X-10-S1-S9

**Published:** 2010-12-13

**Authors:** Manveen Puri, Hassan Masum, Jennifer Heys, Peter A Singer

**Affiliations:** 1 McLaughlin-Rotman Centre for Global Health, University Health Network and University of Toronto, 101 College Street Suite 406, Toronto ON, M5G 1L7, Canada

## Abstract

**Background:**

Biopiracy – the use of a people’s long-established medical knowledge without acknowledgement or compensation – has been a disturbing historical reality and exacerbates the global rich-poor divide. Bioprospecting, however, describes the commercialization of indigenous medicines in a manner acceptable to the local populace. Challenges facing bioprospectors seeking to develop traditional medicines in a quality-controlled manner include a lack of skilled labor and high-tech infrastructure, adapting Northern R&D protocols to Southern settings, keeping products affordable for the local population, and managing the threat of biopiracy. The Malagasy Institute of Applied Research (IMRA) has employed bioprospecting to develop new health treatments for conditions such as diabetes and burns. Because of its integration of Western science and Malagasy cultural traditions, IMRA may provide a useful example for African and other organizations interested in bioprospecting.

**Discussion:**

IMRA’s approach to drug development and commercialization was adapted from the outset to Malagasy culture and Southern economic landscapes. It achieved a balance between employing Northern R&D practices and following local cultural norms through four guiding principles. First, IMRA’s researchers understood and respected local practices, and sought to use rather than resist them. Second, IMRA engaged the local community early in the drug development process, and ensured that local people had a stake in its success. Third, IMRA actively collaborated with local and international partners to increase its credibility and research capacity. Fourth, IMRA obtained foreign research funds targeting the “diseases of civilization” to cross-fund the development of drugs for conditions that affect the Malagasy population. These principles are illustrated in the development of IMRA products like Madeglucyl, a treatment for diabetes management that was developed from a traditional remedy.

**Summary:**

By combining local and international research interests, IMRA has been able to keep its treatments affordable for the Malagasy population. Our analysis of IMRA’s history, strategy, and challenges suggests that other developing world institutions seeking to use bioprospecting to address issues of local access to medicines would be well-advised to treat traditional medical knowledge with respect and humility, share its benefits with the local community, and pursue strategic partnerships.

## Background

Finding a new lead in drug discovery is a tremendous challenge—much like seeking a needle in a haystack. One resource for success in the search for bioactive compounds is biodiversity. Bioprospecting is the commercialization of indigenous medicines in a manner acceptable to the local populace [1]. In addition to promoting economic development of the host country, bioprospecting is often associated with sustainability and the preservation of local biodiversity. Bioprospecting stands in contrast to biopiracy in which local and traditional stakeholders are neither consulted nor benefit from the development of drugs based on local flora and fauna.

In recent years, the efforts of several bioprospecting organizations have led to promising drugs being developed to treat conditions from cancers to malaria [2]. During its 52 year history, the Malagasy Institute of Applied Research (IMRA) has documented the ethnomedical uses of over 6000 plants and produced over 40 plant-based drugs and ‘nutraceuticals’ for local use and export [3]. IMRA’s early achievements are tied to its founder Dr. Albert Rakoto-Ratsimamanga’s development of the diabetes drug Madeglucyl; the discovery and development of this drug provide a microcosm of IMRA’s processes.

We used a case study design. Our analysis is based on interviews with key informants including site visits in Madagascar, and literature analysis. Where not specifically noted or referenced, the report is based on analysis of these interviews. Semi-structured, face-to-face interviews took place in Antananarivo, Madagascar in October 2007. These were followed by email discussions, updates, and feedback with IMRA from August to October 2009. Analysis of transcripts was supported by qualitative data analysis software ATLASti and NVivo. We also analyzed background documents from peer-reviewed literature, news items, reports, and books, as well as reports from the Government of Madagascar, World Health Organization, World Intellectual Property Organization, and the organizational website of IMRA. IMRA was asked to fact-check the case study; the analysis and interpretation is our own. All quotes are from the interviews unless noted, and with permission. This study was approved by the Office of Research Ethics of the University of Toronto.

This article analyzes the history and studies the lessons learned by IMRA, and in doing so suggests how research institutions in the developing world might approach the challenge of making effective use of their biodiversity resources. After discussing IMRA’s approach to bioprospecting, we go on to examine the company’s partnerships and operating strategy. We then consider lessons from IMRA, and close by considering its relevance to the broader question of ethnomedicine’s potential benefit for global health.

## Discussion

### Madagascar: biodiversity threatened by biopiracy

With over 5% of the world’s biodiversity on 0.004% of the world’s landmass, Madagascar is one of the world’s top three conservation hotspots [4-6]. The Malagasy people, however, are the seventh poorest people in the world. Madagascar’s political history, from French colonial rule in the 19^th^ and 20^th^ centuries to a military coup in 2001 and more recent political troubles, has only exacerbated the historic divides within its ethnically heterogeneous population [4,5].

The threat of biopiracy remains prevalent in Madagascar. The Malagasy people reportedly did not share the benefits of the commercialization of the Madagascar periwinkle (*Catharanthus roseus*) into the drug Vincristine by a Northern pharmaceutical company [7].

### From humble beginnings to modern research institute

In 1958, with a personal investment derived from royalties accrued through his previous discoveries, Dr. Albert Rakoto-Ratsimamanga founded IMRA [8]. Being of noble birth, Rakoto-Ratsimamanga was one of the few Malagasy of his generation who was educated abroad, and received doctorates in both science and medicine at the Université de Paris. On his return, Rakoto-Ratsimamanga applied his training to aid his fellow citizens. As a result of a life dedicated to the betterment of those less privileged than himself, Rakoto-Ratsimamanga was honored by his fellow citizens as “Malagasy Man of the 20th century.”

Since Rakoto-Ratsimamanga’s death in 2001, IMRA has been headed by his wife, Suzanne Ratsimamanga. Under her leadership, IMRA has grown into an organization that provides permanent employment for a staff of about 150, and seasonal employment for almost 15,000 rural villagers.

While IMRA’s core purpose is to conduct research and develop and market affordable drugs, the organization also aims to promote Malagasy culture and preserve local biodiversity. According to its management, at the time of the case study IMRA was comprised of four main divisions [9]:

• A department of research and development (R&D). R&D is a non-profit division of IMRA. IMRA also trains the next generation of Malagasy researchers (mainly through collaborations with local and international universities).

• A biodiversity unit that includes a botanical garden and lab for collecting and preserving endangered medicinal plants.

• A department for the production, quality control, distribution, and export of products (including drugs, nutraceuticals, oils, and cosmetics). This division is separately incorporated as Soamadina Limited, and is a for-profit entity.

• A health clinic which offers free health care consultations to local villagers.

### Madeglucyl: a novel diabetes drug

In 1965, Albert and Susan Rakoto-Ratsimamanga discovered that traditional healers were using a novel method to diagnose diabetes: healers asked patients to urinate next to an anthill, and observed the reaction of the ants. While ants avoid the urine of healthy humans, the urine of diabetic patients is rich in glucose and is especially attractive to the insects. After watching healers prescribe a plum-like fruit from the plant *Syzygium cumini* to treat their patients, the Ratsimamangas decided to systematically study this plant.

IMRA faced several challenges along the way. First, IMRA had to limit the local practice of cutting down *Syzygium cumini* trees for fuel and construction material. IMRA addressed this through dialogue that convinced the local villagers that *Syzygium cumini* trees were more valuable for their fruit than their wood.

Designing an effective system of seed collection was another challenge. The seeds, which contain the active medicinal ingredient, have to be harvested and quickly processed during the short peak period of fruit growth. From the beginning, IMRA believed that the rural people who populated the areas where the tree grew were the ones best suited to aid the seed collection process. After a short training period, the local people took quickly to seed collection and drying. Improvements to their supply chain network meant that by 1998, IMRA was able to harvest 20 tons of seed during the short collection season.

IMRA’s efforts resulted in the creation of a commercially produced drug, Madeglucyl (a timeline for Madeglucyl’s development can be found in Table 1). Pre-clinical studies on rats established the efficacy and safety of the drug, and led to clinical trials in Madagascar, Germany, and the United States. In December 1997, Madeglucyl was approved as a licensed medicine in Madagascar; although a clinical trial has been conducted in the US, it is reportedly only available as a herbal supplement there [9].

**Table 1:** Timeline for IMRA and Madeglucyl development

**1958**	Albert Rakoto-Ratsimamanga establishes IMRA
**1965**	Albert Rakoto-Ratsimamanga and Suzanne Ratsimamanga begin to work with local healers
**1970**	IMRA launches its *Syzygium cumini* (Madeglucyl) project
**1984**	Initial license for Madeglucyl is registered in Paris
**1985**	Professors study and experiment with *Syzygium cumini* seeds at IMRA and the University of Paris.
**1993**	IMRA is awarded official status as a foundation by government decree
**1996**	Second license is issued, granting Madeglucyl international recognition
**1997**	New Drug Application for Madeglucyl is filed and approved in Madagascar
**1998**	Total harvest of *Syzygium cumini* seeds reaches 20 tonnes
**2007**	Production capacity for Madeglucyl reaches 20kg/day (5g/package)

Clinical studies conducted by IMRA have shown Madeglucyl to be effective in helping to manage both Type I and Type II diabetes; in Type I diabetes, it reportedly reduces (but does not eliminate) daily insulin requirements [9]. Although Madeglucyl is presently sold mainly in Madagascar, it has recently been launched in the international market under the trade name Glucanol Forte^TM ^[10].

### The drug discovery process at IMRA

IMRA uses a multi-disciplinary approach to drug discovery that represents local adaptation of the pipeline approach used by the pharmaceutical industry. The first step involves conducting surveys with villagers and traditional health practitioners to gather information on indigenous practices to treat a particular condition. Through this, researchers are able to compile a bibliography of plants traditionally used to treat the disease in question. IMRA also obtains the consent of tribal elders and local heads before encroaching into their traditional sphere of authority.

The next step involves the collection and botanical study of promising plants. The chemists on the team then proceed to extract active compounds that may be responsible for the plant’s efficacy. Pharmacologists test compounds of interest using *in vitro* and *in vivo* models [11]. The safety and toxicity of the prospective compound is then studied, followed by pre-clinical and clinical studies. (The protocol IMRA uses for these studies is the one developed in 2002 by the Traditional Medicine Program which is part of the WHO regional office for Africa [12].) Finally, the drug is brought to market, which involves regulatory, financial, and marketing considerations as discussed later.

Due to equipment shortages at IMRA, pre-clinical trials are often done abroad in collaboration with partners. The wait times caused by such an arrangement often prolong the time it takes to bring a promising drug to market, and are a limitation of working in a low-resource setting.

### Working with local culture

While IMRA’s approach appears simple, implementing it has required a nuanced understanding of Malagasy culture. The revered stature of healers in Malagasy society means that ordinary villagers may be unlikely to question their methods, as an inquisitive attitude could be interpreted as an affront to the healer’s authority. Yet IMRA’s consultation process with traditional health practitioners may reveal medicines that the healers believe to be efficacious and benign, but which in reality may be ineffective or even harmful. As Madam Baholy Rafatro, a phytochemist at IMRA, puts it: “Sometimes, the population doesn’t tell the traditional healer that they [still] have a headache...they don’t want to tell it to the traditional healer because they are afraid...they don’t want the traditional healer to [say] that ‘Ah, you don’t have confidence in me? Don’t come to see me after.’”

Understanding the rationale behind IMRA’s approach requires an anthropological perspective of illness. In sociocentric societies such as Madagascar, visiting the traditional healer when ill is itself part of the healing process. While such a visit may facilitate a “hoped for result” (i.e. a cure that has a biomedical basis), it always affects an “expected result” (i.e. a confirmation of the illness which in turn reinforces the worldview of the given society) [13]. Accordingly, the healer’s methods (e.g. always prescribing a tea for any illness) may be eliciting a cure through the placebo effect. While both the healer and the healed may defend the efficacy of the tea, the cure it elicits could be culturally specific and have no biological basis. Thus, bioprospecting requires an understanding of local culture if one is to avoid wasting time and money. According to Mr. Denis, a lab manager at IMRA: “It’s not true that the plants that are [traditionally] used for diabetes [are always] efficacious for diabetes; so we screen [more plants], or go for something else.”

Despite the challenges, researchers at IMRA are convinced of the benefits of scientifically studying Madagascar’s biodiversity. Any phytomedicines developed through such a process are likely to be readily accepted by the Malagasy people who “are used to taking plants” according to Madam Baholy Rafatro.

### Regulatory affairs

In 2002, the Ministry of Health in Madagascar established the Department of Traditional Medicine and Pharmacopoeias to oversee the development of laws and regulations related to Traditional Medicines and Complementary and Alternative Medicines (TM/CAM) [14]. IMRA works closely with the Ministry and appears to view the regulatory establishment as a friend rather than a foe. Nevertheless, IMRA’s CSO Dr. Rasoanaivo suggests that the regulatory climate in Madagascar is not yet mature: “The idea of protection of intellectual property rights is very important or else biopiracy will continue.”

While pharmaceutical drugs are regulated in Madagascar, most traditional medicines are classified as over-the-counter drugs and are thus not regulated. There are, however, regulations in place to ensure that manufacturers adhere to Good Manufacturing Practices (GMPs), and produce herbal drugs according to the information contained in the pharmacopoeias. Despite poor oversight, TM/CAMs command the trust of many Malagasy people.

One regulatory challenge relevant to bioprospecting is potential discontent between modern doctors / researchers and traditional healers. While the former consist of physicians educated in the Western biomedical paradigm, the latter maintain the trust of the Malagasy population, and in recent times have been licensed, which has allowed them to practice backed by the legitimacy of the state. The frustration experienced by physicians was expressed by Madam Baholy Rafatro: “Sometimes people go listen to them [the healers] because traditionally they go listen to them, and they ignore the tests and other advice that the doctor has given them.”

### Partnerships and collaborations: bioprospecting under the CNRS program

According to our key informants, IMRA has actively sought external funding to support bioprospecting activities, notably through European Union (EU) grants aimed at promoting bioprospecting. Table 2 lists some of IMRA’s main local and international R&D partners as of 2009. Along with supporting bioprospecting, these partnerships and collaborations aid in training the next generation of Malagasy researchers.

**Table 2:** IMRA’s main partnerships and collaborations

Partner name	Country	Nature of collaboration
Ministry of Health	Madagascar	Collaboration on regulatory affairs. The Ministry awards IMRA a contract to sell its products to local hospitals.
Sanofi-Aventis (pharmaceutical company)	France	Provides equipment and materials for labs at IMRA, and funding. Laboratory and pre-clinical tests (e.g. structure elucidation of biomolecules). Technology transfer from Paris to Madagascar.
Institut de Chimie des Substances Naturelles, CNRS (National Centre for Scientific Research)	France	Work on a bioprospecting project to bring biological extracts and natural products into drug development. Scientific training of IMRA researchers.
University of Belgium	Belgium	Laboratory and pre-clinical tests. Scientific training of researchers. Funding and equipment.
Università degli Studi di Roma "La Sapienza"	Italy	Phytochemical work on Madagascar plants.
Bayer (pharmaceutical company)	Germany	Assistance to IMRA with exports. Technical expertise.
NAPRECA (National Product Research Network for Eastern and Central Africa)	Regional	NAPRECA is a regional consortium that aims to stimulate the development of natural products research in Africa by coordinating such efforts.
RITAM (Research Initiative on Traditional Anti-malarial Methods)	Global	RITAM is an international consortium of researchers working on traditional medicines for malaria.
WHO (World Health Organization)	Global	Work on a project to screen for medicinal plants to treat malaria.

IMRA’s CSO, Dr Rasoanaivo, described two bioprospecting programs that have been conducted in Madagascar. In July 2004, the *Centre National de la Recherche Scientifique* (CRNS) in France signed a memorandum of understanding with the University of Antananarivo, with the participation of IMRA. Under this bioprospecting project, more than 800 diverse plants were collected since 2004; in August 2009, the project was renewed for three years. Plants were reportedly collected with prior consent of the *Ministère de l’Environnement, des Eaux et Forets et du Tourisme*, and extracts exported for screening after an agreement was signed with the *Direction de la Préservation de la Biodiversité.*

A second project called PHYTOCHIK, funded by the *Centre de Recherche et de Veille sur les Maladies émergentes dans l’Océan Indien* (CRVOI) aims to search the area’s biodiversity for compounds targeting the Chikungunya virus on the Indian Ocean islands. It involves multilateral cooperation between Mauritius, Madagascar (IMRA), Réunion Island, France, and Belgium.

Funding through a third project from the International Cooperative Biodiversity Groups (ICBG) reportedly involved agreements with local villagers, and has aided the development of infrastructure in Madagascar, including the the construction of a new laboratory at the University of Antananarivo and equipment upgrades at a laboratory at the University of Fianarantsoa [15,16]. According to key informants at IMRA, these laboratories will play a role in the extraction of active compounds from medicinal plants in the future.

### Finances and raising capital

IMRA has found raising the capital necessary to sustain its operations to be challenging. This problem is made worse by the lack of funding by the Madagascar government for research activities.

As a result, IMRA relies on revenues obtained from the sales of its products, as well as research funding from international organizations to sustain its operations. According to Mr Nivo Rakotoson, IMRA’s commercialization representative, up to 60% of IMRA’s revenues are generated through exports (mainly to European partners in France, Germany, and Italy), while 40% are obtained from local sales. Exports are essential to IMRA’s viability, as the company makes a profit of up to 50% on exports, while only maintaining a 10-15% margin on local products to enhance local affordability.

While IMRA’s revenues are reportedly growing at a rate of over 8% per annum, it remains reliant on its star product Madeglucyl, which accounts for up to 30% of its product revenues. (See Table 3 for a list of other products.) To support its long-term success, IMRA’s commercial arm, Soamadina, channels 30% of its profits back to IMRA, thereby allowing for capacity-building and investments in research and development.

**Table 3:** Key IMRA products [21]

Product name	Plant species	Purpose	Development Stage
Madeglucyl / Glucanol Forte™	*Syzygium cumini*	Anti-diabetic	In local and international markets
Triterpenes constituents	*Centella asiatica*	Wound-healing agent (treats intense burns, leprous wounds and inflamed ulcers) / Cosmetic ingredients	In market
Tazopsine derivative	*Strychnopsis thouarsii*	Prophylactic anti-malarial	Early stage development
TMM	Plant mixture	Anti-leprosy	In market
ODY VATO	*Hylocereus* genus	Against kidney stones	In market
Dangitsyl	Plant mixture	To treat erectile dysfunction	In market

### Marketing and distribution

IMRA’s marketing strategy is simple: If it works well, it will sell. Because of its status as a research-based organization, most of IMRA’s efforts are focused on drug discovery, and less attention is paid to the commercialization of products. According to Mr Nivo Rakotoson, IMRA’s commercialization representative, the Ministry of Health authorizes IMRA to sell directly to hospitals, and IMRA therefore promotes its products through hospital tours where its representatives communicate with doctors.

IMRA owns a network of over 95 pharmacies throughout Madagascar, which serves as a main distribution channel for its products locally. In smaller villages that lack a pharmacy, IMRA’s products are still usually available at the local “*depots de medicaments*”.

Table 3 lists IMRA’s main products. Despite efforts to keep its products accessible to the Malagasy population, IMRA’s products are still expensive for most villagers, and keeping prices low remains a challenge. Increasing the scale of production and making greater use of automation in its production facilities are two ideas that IMRA representatives discussed which might aid in this effort.

IMRA exports its products to European countries such as France, Germany, and Italy [10]. As well, according to Mr Rakotoson IMRA has “loyal clients” overseas who purchase plant products such as essential oils over the internet. From the interviews, it appears that IMRA’s ability to secure foreign buyers for its products is significantly aided by its status as a one-stop authority on Madagascar’s traditional biodiversity.

### The future

In a sign of international recognition of IMRA, Dr. Philippe Rasoanaivo, IMRA’s CSO, was recently awarded the Sven Brohult Award by the International Foundation for Science (IFS) for his research into traditional plants utilized by healers in Madagascar [17].

IMRA is developing relationships with European companies to build a new production facility where drugs are manufactured according to international standards. IMRA also intends to expand the market for Madeglucyl to other African nations in the next few years, and has expressed interest in working with other African national health authorities to create commercial links.

Other phytomedicines are being developed by IMRA, as shown in Table 3. Madecassol, a wound-healing agent developed from *Centella asiatica*, is used to treat burns, ulcers and leprous wounds. Researchers at IMRA are also working on an anti-malarial compound, tazopsine. Derived from the bark of *Strychnopsis thouarsii*, tazopsine has been used for years as an ingredient in a herbal tea remedy for malaria. Tazopsine works by targeting the early stages of malarial infection, decreasing the chance of resistance developing [18]. Researchers at IMRA are hopeful that laboratory tests on tazopsine variants will produce one that is of low toxicity, and hence suitable for human clinical trials.

While IMRA possesses an international outlook, it continues to pay attention to developing treatments for conditions that are widespread in the Malagasy population, such as malaria, pulmonary problems, and diarrheal diseases. As Mr Guy Rakotoson, IMRA’s Head Administrator puts it, “It’s really the philosophy of the foundation to be at the service of the Malagasy population.”

### Lessons learned

IMRA was designed with Madagascar in mind. Where it hit roadblocks and challenges, it found solutions grounded in local realities and constraints. Here, we outline lessons from IMRA’s experience that illustrate how Southern innovators may utilize indigenous biodiversity to pursue pharmaceutical development.

#### Work with local culture

Much of IMRA’s success comes from respecting Malagasy traditions and cultural norms, and using rather than resisting them. Themselves Malagasy, IMRA’s researchers understand local customs. An appreciation of the host country’s culture is important if problems, such as the tension between modern physicians and traditional health practitioners in Madagascar, are to be anticipated and resolved. Bioprospectors must also be careful to scientifically validate local practices if they wish to avoid wasting resources investigating false leads.

On the health delivery side, in medically pluralistic societies such as Madagascar where over 80% of the population uses traditional medicines as a first source of treatment, local populations may more readily accept phytomedical cures that are cheap, non-invasive, often already trusted, and easy to effect in a low-resource setting.

While “work with local culture” is an easy phrase to agree with, the process of local adaptation can be extremely difficult to execute. Part of IMRA’s success in this regard seems to have come from Albert Rakoto-Ratsimamanga’s combination of foreign training and experience with deep local knowledge, which may suggest the value of leadership which is intimately familiar with relevant cultures. Finding such motivated and cross-trained leadership is one barrier to adopting an IMRA-like approach elsewhere; others include cultivating an attitude of humility toward traditional medicine while being grounded in solid science, and the tendency for traditionalists to reject biomedicine.

#### Engage and share benefits with local communities

Southern innovators may wish to engage the local community early in the drug development process. Many of IMRA’s challenges, such as its initial difficulty in establishing a supply network of traditional plants and seeds and its efforts to combat biopiracy, can be seen as different facets of the single problem of local adaptation. A single strategy—bringing the local population into the organization as part of its discovery, harvesting, and distribution systems—successfully addressed all of these concerns and helped bridge the divide between ethnomedicine and biomedicine in conservative Malagasy society.

It is also advantageous for innovators to ensure that locals have a stake in their company’s success, and vice versa. IMRA was not gifted with the same markets as North American or European pharmaceutical organizations; it had to make its products available to a poor population suspicious of Western medicine. Through its benefit-sharing practices that help local villagers to profit from the organization’s success, IMRA claims to have created a sense of solidarity between itself and the Malagasy populace, while at the same time building a strong relationship with its local customer base.

#### Use partnerships for capacity-building and credibility

It is beneficial to collaborate with local and international partners who have complementary strengths. IMRA was challenged by delays in its research caused by the low-resource base of Madagascar, and at the same time faced a shortage of skilled personnel. Through its partnerships, IMRA increased its credibility and research capacity, and benefited from a transfer of research dollars from the global North to the global South.

Poor infrastructure and lack of access to resources common in the West remain key challenges associated with working in a developing country. Despite these challenges, IMRA aims to keep itself relevant by investing in both its equipment and its people. One reason why foreign universities and corporations have been willing to cooperate with IMRA is because it has conformed to internationally accepted standards of science.

The IMRA case illustrates the need to invest in human capital by training researchers through international collaborations and studies. This “capacity-building” may prove costly in the short-term, but is vital in the long-term to ensure that local scientists possess the ability to conduct significant research. However, despite such efforts the number of skilled scientific personnel in Madagascar is limited, and IMRA has experienced difficulty filling some of its positions.

#### Leverage both local strengths and global markets

Southern research institutions can leverage their low-resource setting as a strength. Being situated in Madagascar, IMRA is able to market itself as a local authority on the island’s biodiversity. IMRA’s research capacity and reputation makes it an appealing partner for Northern groups seeking to preserve and sustainably utilize the world’s biodiversity. This strategy demonstrates the potential of adapting research and business methods to local settings, rather than necessarily trying to change the local situation to fit an international market.

In IMRA’s case, the profits generated by exports help keep its products affordable for the local populace, which thus benefits from IMRA’s presence through both access to medicines and increased economic opportunity. While IMRA’s focus is local and it remains committed to research that will lead to the development of affordable drugs for Madagascar, the organization also looks for foreign research dollars. Researchers at IMRA have been able to successfully combine local and international research interests. Other institutions and organizations in similar circumstances may consider coupling funds for projects aimed at combating the “diseases of civilization” with research into conditions prevalent in the South.

## Summary

This article has analyzed the history, challenges, and lessons of IMRA, and in doing so suggested how research institutions and countries in the developing world might make effective use of their biodiversity resources. Because of its integration of Western science and local cultural traditions, IMRA may provide a useful example for African and other organizations interested in bioprospecting. It may also be worth considering the value of having a relatively well-resourced national organization to facilitate development and production of compounds from local biodiversity resources.

As countries from Cameroon to Costa Rica harness their biodiversity and ethnomedical knowledge to propel their economies into the 21^st^ century [2], the need for a holistic approach to bioprospecting, one that carefully considers the interest of all stakeholders, will only grow. This is particularly relevant in the African context where ethnomedical knowledge has traditionally been passed down from generation to generation through the oral tradition [19]. The lack of written records and the presence of rare medicinal plants on privately owned lands has limited foreign access to ethnobotanical information, making cooperation with local actors all the more necessary.

IMRA has aimed to adopt an approach to scientific research that integrates health care, biodiversity conservation, and production. In the course of its work, IMRA has advocated practices that protect the environment, respect local culture, and empower local populations to share in the economic benefits of bioprospecting. IMRA’s relationship to traditional Malagasy healing has been important to the organization’s achievements to date.

Northern organizations seeking to bioprospect in the global South should be aware of past biopiracy incidents, and of the risk of creating new power differentials and exacerbating existing ones in post-colonial African society [20]. Stoked by identity politics and a broader debate about the degree to which traditional knowledge should be protected, a number of local actors have called for increased protection. In reality, the middle ground as represented by organizations like IMRA between complete protectionism and unfettered access to ethnomedical knowledge may represent the best hope of pushing forward the boundaries of medical research [21-23].

One goal of this analysis of IMRA has been to help inform those who wish to start, partner with, invest in, or simply better understand organizations in the developing world that engage in bioprospecting to tackle global health challenges. Given the potential of traditional medicines to address issues of local access to healthcare, it is vital to address barriers to their development, as well as to identify good practices and potential policy interventions to mobilize the developing world’s pharmaceutical potential.

Organizations may wish to consider the principles discussed in this article: treating traditional medical knowledge with respect and humility, sharing its benefits with the local community, developing a collaborative research and funding base, and translating research into appropriate health products with local impact. In doing so, organizations may increase their chances of bridging the divide between ethnomedicine and biomedicine in order to develop new health treatments.

## Competing interests

None declared.

## Authors' contributions

MP, HM, JH, and PAS contributed to the concept and design of this study, analyzed the findings, and participated in manuscript development.
